# Long-Term Medical Follow-Up (for More than 15 Years) of a Patient with Stage IA Mycosis Fungoides Originally Presenting in Childhood: Remission for >15 Years with Localised Electron Beam Therapy

**DOI:** 10.1155/2021/5541246

**Published:** 2021-03-13

**Authors:** Eric Bessell, Martin Dalton, John David Parry

**Affiliations:** ^1^Department of Clinical Oncology, Nottingham City Hospital, Nottingham, UK; ^2^The Surgery, Ravenshead, Nottinghamshire, UK

## Abstract

A man now aged 80 years has received specialist care for stage 1A mycosis fungoides for 58 years. The disease developed in childhood. Long-term follow-up (>30 years) of patients with mycosis fungoides is infrequently described in the world literature. The disease in this patient was limited to 5 areas, but these were large (up to 25 cm in diameter). The rest of the skin was normal clinically. All 5 areas were treated separately with electron beam therapy (3–4 MeV) to a dose of 30 Gy in 15 fractions over 3 weeks between 2000 and 2005. Complete regression was obtained in all 5 areas, and the patient has been in complete remission for 15 years after living with the disease previously for over 40 years.

## 1. Introduction

Mycosis fungoides (MF) is the most frequent type of primary cutaneous T-cell lymphoma but is uncommon with an annual incidence of 4 cases per million [1]. The average age at diagnosis is 59 years [2], and it rarely occurs in children [3]. There is therefore little opportunity to follow patients for very long periods. Long-term follow-up has been reported by the Italian Study Group for Cutaneous Lymphoma [2]. Twenty-seven Italian centres were involved in reporting follow-up information on patients treated between 1975 and 2010 with MF, and the median follow-up was 14.5 years (range: 1–35 years). The 10-year survival rate for early-stage disease (1A) was 93%, and the disease progression at 30 years was 13%. Patients in this study with stage 1A/1B disease had an annual rate of progression to stage IIB disease of 1–2% which was maintained for 15 years from diagnosis. Long-term follow-up of MF and Sezary syndrome has also been reported from Stanford University School of Medicine in the USA [4]. They reported on 525 patients treated between 1958 and 1999. The 30-year overall survival for 155 patients with stage 1A MF was 54%, and the disease-specific survival for these patients was 98%. The annual rate of progression to more advanced disease was similar to the Italian study (1–2% for 15 years).

## 2. Case Report

A man now aged 80 years presented with a rash in childhood (at the age of 3 years, in 1944, according to his mother). All correspondence and clinical notes relating to MF since 1962 have been reviewed by the authors. The rash remained unchanged according to the patient, and no specific diagnosis was made until 1962 when he was 21 years old. A diagnosis of parapsoriasis [5] was then made by a dermatologist, but the skin condition was not described in detail; it was called a long-standing skin eruption. He was treated with triamcinolone in Eucerin (1 in 10) ointment. The rash remained unchanged until 1971 when the patient was described as developing raised infiltrated lesions particularly involving his upper left thigh. A biopsy was then taken which showed early MF, and he was treated with betamethasone ointment. Again, there was no significant change in the patches/plaques, and when systemic PUVA was introduced in Nottingham in 1982, he received a short course of PUVA which resulted in intense irritation of the involved skin. He continued with betamethasone until this reaction resolved. In 1991, a further biopsy confirmed early MF, and he was treated with clobetasone cream intermittently for the next 9 years. During that time, the plaques on his left wrist and left inguinal region became more prominent.

**Figure 1:**
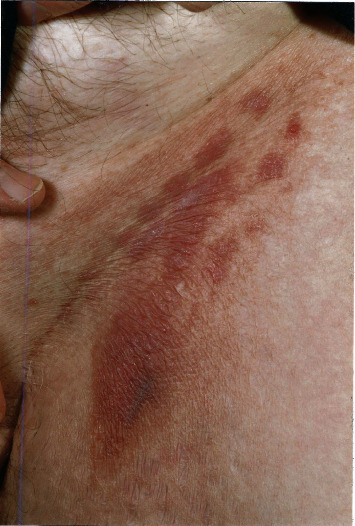
Multiple plaques of mycosis fungoides in the skin of the left inguinal region.

**Figure 2:**
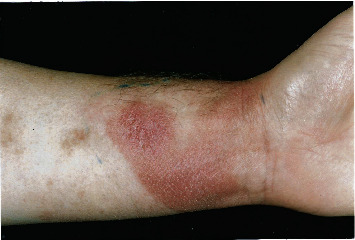
Plaque of mycosis fungoides on the anterior left wrist.

In 2000, he was referred by a dermatologist to an oncologist for consideration of localised electron beam therapy because of progression of MF in the skin of the left wrist and left inguinal region. On examination, there were multiple plaques in the skin of the left inguinal region 15 × 15 cm in overall dimension (Figure 1) and a plaque 8.6 × 6.0 cm on the anterior left wrist (Figure 2). In addition, there was a large (25 × 15 cm) area on the lateral aspect of his right thigh which had not changed in recent years, a 3 cm area on the lateral aspect of his left thigh, and a patch 13 × 8 cm in the posterior left thigh extending down to the popliteal fossa. There were, therefore, a total of five areas of skin involvement.

There were no visible areas elsewhere in the skin, and no lymph nodes were palpable. A skin biopsy in the left inguinal region showed a hyperkeratotic, parakeratotic, acanthotic epidermis with the superficial dermis bearing a dense lymphoid infiltrate of predominantly T-cells (CD 34+, CD4+, and CD8−). The MIB1 labelling index was low, but a clonal T-cell receptor gene arrangement was demonstrated consistent with MF. The stage was IA with less than 10% of the skin involved (TNM staging: T1b) [6].

A treatment strategy was discussed with the patient, and the plan was to treat the most active areas (left wrist and left inguinal region) first to establish whether a complete response could be obtained rather than treating all five areas at the same time. Total skin electron beam therapy was considered, but the MF had been limited to defined areas for many years.

Therefore, in November 2000, electron beam therapy (4 MeV) was given to the skin of the anterior left wrist (30 Gy in 15 fractions over 23 days), and in January 2001, the same treatment was given to the skin of the left inguinal region. Complete regression of both these areas of MF was achieved. A biopsy of the area on the lateral aspect of the right thigh in March 2001 confirmed patch stage MF, and progression of this disease in the skin of the right thigh was observed during 2001. The large area increased in size with new adjacent lesions developing with soreness as a prominent symptom. The whole area in the lateral right thigh was treated with electron beam therapy in the same way as the previous areas in the left inguinal region and left wrist. Again, complete regression was achieved in the skin of the lateral right thigh. The remaining areas of patch disease in the lateral and the posterior left thigh were observed over the next 4 years, and no significant change was seen. The area in the skin of the lateral left thigh had been present since the 1960s. A decision was then made with the patient in 2005 to treat both these areas (the lateral and the left posterior thigh) so that all visible diseases would have been treated (there had been no recurrence of the three previously treated areas of MF). Electron beam therapy (2.7 MeV) was given in October 2005 to both areas (30 Gy in 15 fractions over 21 days), and again, complete remission was obtained in the skin of the lateral and the left posterior thigh.

In 2020, he remained in complete remission from MF with no recurrence of any of the five treated areas and no new lesions developing. After living with MF for over 40 years, he has now been in remission for 15 years.

## 3. Discussion

A case report demonstrating over 50 years of follow-up and treatment for MF has not been published in the world literature before presumably because MF is rare in childhood. There is considerable stress associated with living with cutaneous lymphoma for decades and much relief when complete remission is achieved [7]. This case shows that long-term remission can be achieved with IA disease as has been shown in the Italian and American long-term studies. The key decision is when to start definitive treatment with electron beam treatment.

The British Association of Dermatologists and UK Cutaneous Lymphoma Group guidelines for the management of primary cutaneous lymphomas were published in 2018 [8]. No guidance is given addressing the age at which treatment with electron beam therapy should be started. In the guidance, it stated that MF is a multifocal disease, and whilst local control with radiotherapy is readily achievable, this is usually a palliative approach. In addition, it was stated that rarely, MF can present as a solitary patch or plaque, and local radiotherapy can be used with curative intent (20–30 Gy in 2 Gy fractions). This dose (30 Gy in 2 Gy fractions) was used for the patient described in this case report with curative intent but to 5 areas rather than one. The European Organisation for Research and Treatment recommendation (2017) is 20–24 Gy in this situation [9]. The patient in this case report has been in remission from MF for more than 15 years.

There should be a detailed discussion with patients with stage IA MF, whose disease involves more than one site, but has not changed over many years, about the possibility of potentially curative treatment with localised electron beam therapy. This discussion could have been started when the patient was in their 40s or 50s rather than at age 60 years as in this case if the curative potential of radiotherapy was better understood.

## 4. Conclusion

Localised electron beam therapy can be used with curative intent in stage IA MF with more than one lesion if the multiple patches and plaques have been unchanged for several years.

## Data Availability

This case report is entirely based on the detailed clinical records held by all the three authors. No data were associated with this report which could be added as the supplementary material.
